# Overview of Geriatric Trauma in an Urban Trauma Center in Eastern China: Implications from Computational Intelligence for Localized Trauma-Specific Frailty Index System Design

**DOI:** 10.1007/s44196-023-00247-0

**Published:** 2023-04-18

**Authors:** Sheng Dong, Tie Wu, Yi-Feng Wu, Zu-Liang Min, Ming-Yu Xue

**Affiliations:** Department of Emergency Surgery, Wuxi No. 9 People’s Hospital Affiliated to Soochow University, 999 Liangxi Road, Wuxi, People’s Republic of China

**Keywords:** Geriatric trauma, Epidemiology, Quality improvement, Trauma-Specific Frailty Index

## Abstract

Due to the lifestyle and activity of the aging population, it is expected that geriatric trauma will increase and become one of the major challenges in health care. The objective of this study was to determine epidemiological differences between geriatric trauma patients and their younger counterparts, and to find the implications for localized Trauma-Specific Frailty Index (TSFI) system design. This study was a retrospective analysis of adult patients registered in the Trauma Registry, comparisons were made between the geriatric patients, aged over 65 years old, and the younger patients, aged 18–64 years old. Variables were collected include demography, injury mechanism, type, severity of injuries sustained, and outcomes. From July 2018 to July 2021, 2594 trauma patients were evaluated. Injury severity score (ISS) in the geriatric patients’ group is not higher than the younger patients’ group statistically (*P* = 0.066), and results in increased ICU occupancy and mortality risk as compared with the younger patients (*P* < 0.05). The majority of geriatric patients suffered falls from low heights and traffic accidents. The geriatric patients most suffered isolated injuries of the extremities/pelvis (31.8%) commonly. In contrast to the younger patients, they had more injuries of the head/neck or polytrauma (*P* < 0.05). This study provided a snapshot of the trauma burden in a proportion of the urban geriatric patients in Eastern China. The geriatric patients are unlike their younger counterparts, and their unique features should be considered in the future development of computational intelligence, particularly in the case of localized TSFI system design.

**Supplementary Information:**

The online version contains supplementary material available at 10.1007/s44196-023-00247-0.

## Introduction

Trauma is one of the leading causes of disability and death worldwide [1]. It is estimated that 5.8 million people around the world die from trauma every year, accounting for about 10% of the global death tolls [2]. Rapid urbanization and industrialization in low- and middle-income countries (LMICs) have shifted the focus of the global burden of disease to trauma [3]. Trauma remains to be one of the greatest global public health challenges of our time.

The progress of medical care has brought unprecedented growth of life expectancy in the world [4]. Aging is now a phenomenon worldwide, with the population aged 65 and over projected to increase from 9.3% in 2020 to 16.0% in 2050 [5]. In addition, elderly people's lifestyles are becoming more and more independent and active [6]. As a result, a growing number of elderly people sustain severe injuries in accidents [7–9]. Recent studies shown that patients over 65 years old have accounted for the fifth place of all trauma admissions and trauma is the fifth leading cause of death among this group [10]. The trauma mechanisms and injury patterns in geriatric patients are obviously different from those seen in younger patients [11, 12]. The phenotype of frailty is more common in geriatric patients and related to a higher trauma frailty index [13–15]. Frailty is defined as a biologic syndrome of decreased reserve and resistance to stressors, which means adverse outcomes [16]. The presence of multiple comorbidities in elderly people and overall decreased physiological reserve, clinical decision-making often becomes challenging.

The important thing is to better understand the characteristics of geriatric trauma patients, so as to anticipate their needs and resource utilization. While many trauma scores have been put forward over the years, there is no consensus on which assessment tools is superior, as these scores were developed in different settings and targeted different subsets of the geriatric population. There are few studies that comprehensively describe the injury patterns, socio-demographic data and patient outcomes of the geriatric trauma patients. Most of the published documents on trauma focus on a specific type of injury, such as a traumatic brain injury or injury mechanism [17, 18]. People know little about the local trauma. This research aims to provide quantitative data to fill this void. We described the situation of an urban trauma center in Eastern China, and evaluated the age-related injury patterns and trauma mechanisms. There is still no gold standard for the current assessment methods of frailty. The optimum frailty assessment in geriatric trauma patients is still controversial. The purpose of this study is to determine the epidemiological differences between the geriatric trauma patients and the younger counterparts, and to find the implications for localized TSFI system design and develop machine and deep learning methods for monitoring geriatric patients in the field of healthcare informatics.

## Methods

### Patients

Wuxi is the third largest city in Jiangsu Province and one of the important cities in the Yangtze River Delta region in eastern China. Wuxi city has a population of over 7 million. People over 65 years old make up 15% of the city’s population. Trauma patients in Wuxi were mainly transported to one of five tertiary hospitals (trauma centers). Wuxi No. 9 People’s Hospital is an academic institution and trauma center of Wuxi city. A retrospective observational study was conducted by using the data from the Trauma Registry of Wuxi No. 9 People’s Hospital. Data were gathered from patients who presented to the emergency departments from July 2018 to July 2021. The enrollment criteria for all registrations are trauma patients who present within 24 h of injury, or patients who die within 24 h of admission.

Ethical approval was waived by the Wuxi No. 9 People’s Hospital Ethics Committee as the observational nature of this study. Individual consent has been waived by the ethics committee. All ongoing operations were part of the routine. This study was conducted under the ethical standards of the Declaration of Helsinki (as revised in 2013).

Despite the increasing importance of geriatric trauma in medicine, there is no universally accepted definition. Several clinicians use the age threshold as a recognized distinction. We defined the geriatric patients as those over 65 years old, based on the practice of existing literature [19–21].

### Data Collection

This study was a retrospective analysis of adult patients (age ≥ 18 years old) registered in the Trauma Registry, comparisons were made between the geriatric patients, aged over 65 years old, and the younger patients, aged 18–64 years old. Variables were collected include demography, injury mechanism, type, severity of injuries sustained, and outcomes. Patient suffering from any potentially form of trauma, such as traffic accidents (pedestrian, pedal cycle, motorbike and car collisions), physical violence (assault or self-mutilation by fist, blunt object and stabbings), low and high falls or industrial accidents, were included.

The severity of each injury was measured in line with the Abbreviated Injury Scale (AIS)-2005, ranging from 1 (minor) to 6 (fatal) [22, 23]. We used AIS to determine whether the patient suffered severe trauma in the six regions of body (including head/neck, face, chest, abdomen, extremities/pelvis, and external). We defined major trauma as patients with an ISS higher than 15, a non-major injury with an ISS lower than 15. Types of injuries were categorized into blunt injuries and sharp injuries (including stab, cut, and chop wounds). Patients with both sharp and blunt lesions in the same anatomical region were considered to be sharp lesions. Multiple injuries or polytrauma in the same anatomical region were also regarded as only one injury to the corresponding anatomical region. Although injury types are not directly reported in this study, each body region includes all types of injuries such as fractures, dislocations, contusions, lacerations, abrasions, burns and so on.

### Statistical Analysis

A chi-square test was adapted for comparison between two groups (or Fisher’s exact test, when appropriate). The parameter variables were tested by Student *t* test. The SPSS software was used for all statistical analyses. (SPSS Inc., Chicago, IL, USA, version 24.0). A two-tailed, with *P* < 0.05 is considered statistically significant.

## Results

### Demographic Characteristics

From July 2018 to July 2021, 2594 adult patients were included in the trauma registry. There were 1707 males and 887 females, with an average age of 50.6 years. The youngest patient was 18 years old and the oldest patient was 105 years old. 81.1% (*n* = 2103) were aged 18–64 years old, and 18.9% (*n* = 491) were aged 65 years old or above. Among the younger patients (18–64 years old), the median age is 47 years old, IQR 36–54 years old and 1433 (68.1%) were male; while in the geriatric patients (≥ 65 years), the median age was 72 years old, IQR 68–80 years old, and 274 (55.8%) were male.

The severity of injury (median ISS 8, IQR4–9) was low, and patients with major trauma (ISS > 15) only account for 10.8%. ISS in the geriatric patients’ group is not higher than the younger patients’ group statistically (*P* = 0.066). Among the younger trauma patients, injuries were mainly secondary to blunt trauma (74.4%), while sharp trauma comprised a minority of presentations (25.6%). Blunt trauma was much higher than sharp trauma in the geriatric patients with 441 cases (90.0%) overall. There were statistically significant differences in the number of patients admitted to ICU, 5.5% (27/491) of geriatric trauma patients, and 3.3% (69/2103) of younger trauma patients (*P* < 0.05). 1.5% (39/2594) patients who died within 24 h of admission, 2.6% (13/491) of geriatric trauma patients, and 1.2% (26/2103) of younger trauma patients (*P* < 0.05). The clinical characteristics and demographics of all the patients are shown in Table 1.

**Table 1:** Demographic and injury event details

Characteristics	Total (*n* = 2594)	Younger patients (*n* = 2103)	Geriatric patients (*n* = 491)	*P*
Demographic data				
Gender, *n* (%)				** < 0.001**
Female	887 (34.2%)	670 (31.9%)	217 (44.2%)	
Male	1707 (65.8%)	1433 (68.1%)	274 (55.8%)	
Age, years				
Mean ± SD	50.6 ± 16.2	45.0 ± 11.8	74.6 ± 8.4	** < 0.001**
Injury-related data				
Trauma type, *n* (%)				
Blunt	2004 (77.3%)	1563 (74.4%)	441 (90.0%)	** < 0.001**
Sharp	587 (22.7%)	538 (25.6%)	49 (10.0%)	
Injuries sustained, *n* (%)				** < 0.001**
Isolated injury	1777 (68.5%)	1474 (70.1%)	303 (61.7%)	
Multiple body regions	817 (31.5%)	629 (29.9%)	188 (38.3%)	
Injury severity score				
ISS, (median, IQR)	8 (4–9)	8 (4–9)	5 (4–9)	0.607
Major trauma (ISS > 15), *n* (%)	279 (10.8%)	215 (10.2%)	64 (13.1%)	0.066
Admission to ICU, *n* (%)	96 (3.7%)	69 (3.3%)	27 (5.5%)	**0.019**
Death within 24 h, *n* (%)	39 (1.5%)	26 (1.2%)	13 (2.6%)	**0.021**

Bold values indicate the level of statistical significance was *P* < 0.05

**Fig. 1:**
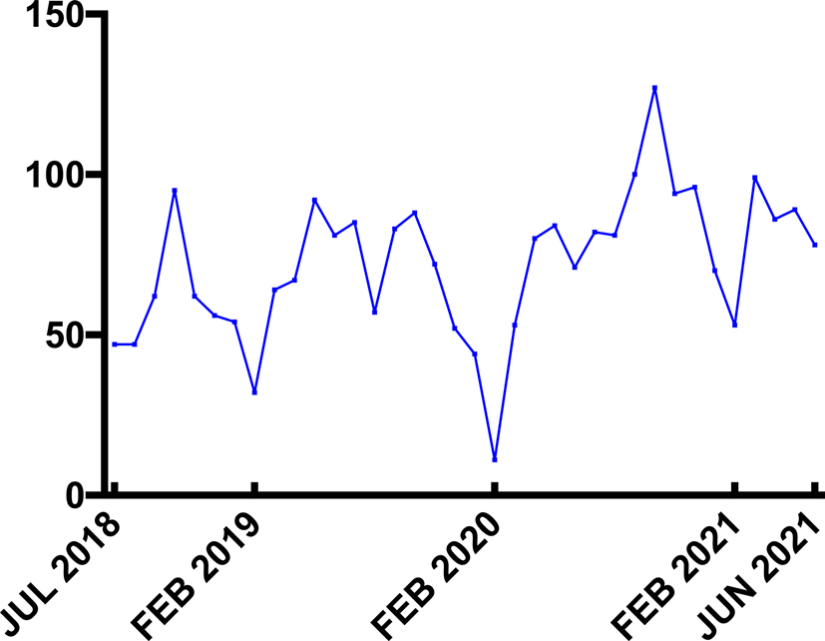
Monthly distribution of trauma center admissions

Being a country with low epidemic situation in COVID-19, China's social and health-care systems have not gone through the overwhelming impact as the rest of the world [24]. In terms of the number of hospital admissions per month, the most substantial hit occurred in the first 2 months of the lockdown. Coincidentally, the same month was also the lowest in the research period, because of the Spring Festival. However, as the blockade continued, the number of trauma center admissions gradually started increasing until reaching as usual (Fig. 1). Basic demographic characteristics of the pre-COVID-19 and COVID-19 cohorts are reported in Supplementary Table 1.

### Trauma Mechanisms and Injuries

As shown in Table 2, the geriatric patients most suffered isolated injuries of the extremities/pelvis (31.8%) commonly. In contrast to the younger patients, they more commonly presented with injuries of the head/neck (18.5% geriatric patients versus 10.2% younger patients, *P* < 0.05). Patients in two age groups presented with a similar percentage of other isolated injuries (face, chest, abdomen and external). In geriatric patients, polytrauma was the most frequent trauma (38.3% geriatric patients versus 29.9% younger patients, *P* < 0.05).

**Table 2:** Comparison of injured anatomical regions by patient population

Anatomical region	Younger patients (*n* = 2103)	Geriatric patients (*n* = 491)	*P*
Isolated injury			
Head/neck	215 (10.2%)	91 (18.5%)	** < 0.001**
Face	27 (1.3%)	5 (1.0%)	0.631
Chest	91 (4.3%)	24 (4.9%)	0.587
Abdomen	40 (1.9%)	6 (1.2%)	0.304
Extremities/pelvis	1023 (48.6%)	156 (31.8%)	** < 0.001**
External	78 (3.7%)	21 (4.3%)	0.554
Multiple body regions	629 (29.9%)	188 (38.3%)	** < 0.001**

Bold values indicate the level of statistical significance was *P* < 0.05

**Fig. 2:**
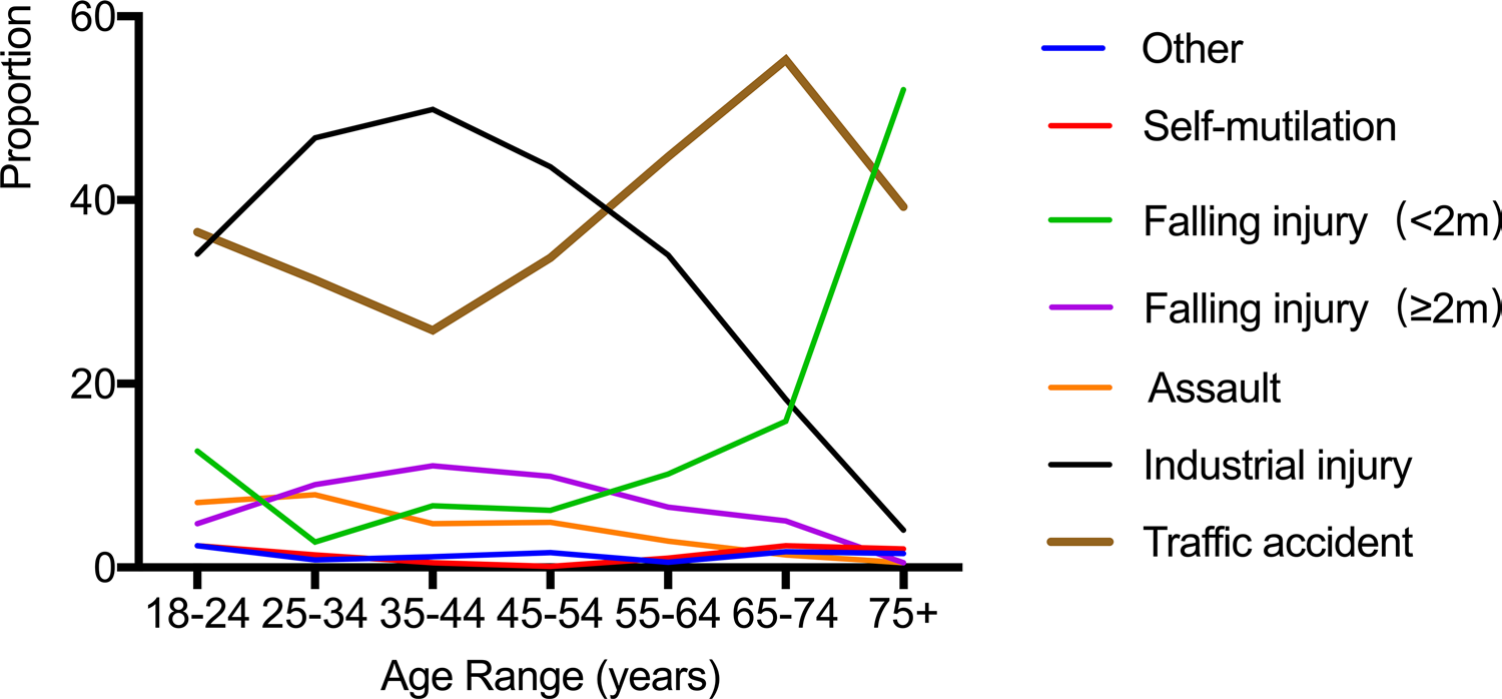
Trends of injury mechanism in different age groups

Among the geriatric patients, traffic accidents accounted for the majority of injuries (48.9%), 30.3% of patients were injured by falls of < 2 m, and 3.3% of patients were injured by falls of ≥ 2 m. Industrial injury (12.6%), self-mutilation (2.3%), assault (1.0%) and other accidents (1.6%). On the other hand, the younger patients have heterogeneous injury mechanisms. Industrial injury accounted for 42.5% of presentations, traffic accident 34.6%, assault 5.0%, falling injury (≥ 2 m) 8.9%, falling injury (< 2 m) 7.1%, self-mutilation 0.8%, and other accidents (1.2%). The mechanism of injury in the geriatric patients and the younger patients are reported in Table 3. All of the patients suffered mostly from road accidents. In comparison, the younger patients were found to be more likely involved in industrial injuries. In the group of the geriatric patients, falling injury (< 2 m) was the second most common cause of injury. The mechanism of injury is shown in Fig. 2 by the different age groups. Their trends were also summarized.

**Table 3:** Comparison of mechanism of injury by patient population

Mechanism of injury, *n* (%)	Younger patients (*n* = 2103)	Geriatric patients (*n* = 491)	*P*
Traffic accident	727 (34.6%)	240 (48.9%)	** < 0.001**
Industrial injury	893 (42.5%)	62 (12.6%)	** < 0.001**
Assault	106 (5.0%)	5 (1.0%)	** < 0.001**
Falling injury (≥ 2 m)	187 (8.9%)	16 (3.3%)	** < 0.001**
Falling injury (< 2 m)	149 (7.1%)	149 (30.3%)	** < 0.001**
Self-mutilation	16 (0.8%)	11 (2.3%)	**0.004**
Other^a^	25 (1.2%)	8 (1.6%)	0.433

Bold values indicate the level of statistical significance was *P* < 0.05

^a^Includes electrical, chemical, recreational, iatrogenic injuries and so on

## Discussion

Trauma present a significant public health problem in LMICs [25, 26]. In the past three to four decades, the acceleration of urbanization and industrialization has led to an alarming increase in the accidental injury rate [27]. At the same time, life expectancy has increased significantly in recent decades, the population continues to age [28]. With the extension of life span, the improvement of the mobility and activity level of the elderly population, they also face more harm. Trauma centers are dealing with more and more injured elderly patients [29]. The objective of this study is to identify the epidemiology of elderly trauma patients in modern city. Several differences have been identified regarding trauma mechanism, injury pattern, diagnosis performed and results.

The first important finding is that the injury pattern of the elderly patients has changed. Evaluation data show that the elderly are more often admitted to the hospital because of traffic accidents and falls from lower heights, and results in increased ICU occupancy and mortality risk as compared with the younger patients. This was also described in several other studies. It has previously been reported that older adults are at increased risk for traffic accidents due to visual and auditory impairment [30]. Meanwhile some geriatric patients lack safety awareness of the risk of traffic accidents. In many Asian countries, motorized two-wheeled vehicles (motorcycles and scooters) are the first choice of low-cost and high-mobility vehicles for commercial and personal use, resulting in high road injury rates [31]. In addition, rapid urbanization coupled with a lack of proper traffic regulations leads to the increasing burden of disease related to road injuries and road fatalities. In a fast urbanizing world, it is necessary to know how urban and transportation planning and design decisions affect the health of the elderly [32]. We found that most of the falls occurred from a standing height (< 2 m) and incidence of falls grew with increasing age. The rise in the incidence of falls with aging is caused by impairment of the visual, cognitive, neurological, musculoskeletal and cardiovascular systems [33]. Therefore, fall prevention programs should attempt to identify such risk factors and treat them when possible. Public education, nursing staff training and family safety awareness for preventing falls should be carried out at the community level to reduce such avoidable accidents. Falls in geriatric patients may be a pre-frail indication and may be a signal of underlying balance or vision disorders. Among other strategies, early intervention by using effective exercise programs to train proprioception, muscle strength and balance has been proved to reduce the risk of falls in such patients.

Notwithstanding, the issue of assault and self-mutilation is way too broad for the scope of this single-center study. We could only conjecture the possible explanation of our statistics from the above data, but not jump to a firm conclusion. Although the number of samples is small, the incidence of self-mutilation behaviors increased dramatically in the geriatric patients with 2.3% admissions, whereas there were only 0.8% self-mutilation admissions in the younger patients. Further analysis of pre-existing mental illness may help to implement targeted interventions in patients at risk and plan appropriate preventive measures, especially among the geriatric population [34].

According to the data reported, orthopedic injuries (extremities and pelvis) were the most common in the elderly. This indicates that it would be useful to assess the availability of orthopedic providers in each institution. Determining this injury burden can provide support for the team to lobby the Health Committee to provide more funds and better distribution of resources. In the meantime, they were also suffering from severe, potentially life-threatening trauma. In particular, the incidence of (isolated) head injury increases with age. On the contrary, the incidence of head injury in the control group was lower, while the incidence of orthopedic injuries (extremities and pelvis) was higher. The priority of the trauma team should be to focus on the treatment of head trauma. Firstly, there is evidence that head trauma can lead to poor outcomes in geriatric trauma patients [35]. In addition, the geriatric patients’ group were more likely to suffer from coagulation disorders due to pre-existing drugs. Previous studies have shown that geriatric patients with traumatic brain injury, especially those who use anticoagulants, have higher mortality rates [36]. Trauma teams should always be alert to possible head injuries in geriatric patients, especially those treated with blood thinners [37]. The overarching goal would be to improve the quality of patient care for the geriatric population. Knowing the injury patterns and their prevalence rate could allow us to petition for expanding the coverage of medical care to include these necessary resources in the special-needs population.

After the COVID-19 outbreak, strict measures to curb the spread of COVID-19 virus, curfews and quarantines were imposed in different degrees all over the world, which was the most severe restrictions on social life in recent times [38, 39]. The data of our research shows that the aforementioned strict restrictions have led to the decrease of the expected rate of trauma admission. As time went by, however, the number of trauma admissions began to increase until it reached the normal value. Just as Leichtle et al. said, it can be explained by "blockade fatigue" coupled with the gradual easing and liberal interpretations of the subsequent administrative orders [40].

Due to the dual effects of frailty and the severity and complexity of trauma, there are some differences among geriatric trauma patients in a certain degree. The early identification and evaluation are critical to optimizing outcomes in geriatric trauma patients. Several frailty measurement tools have been developed and validated in different trauma patient cohorts. However, there is no consensus regarding the criteria of a universal frailty score. In the present study, the characteristics of traumatic injuries were evaluated, identification of frailty features of a geriatric trauma patient should be targeted as a focused area in which improvements could significantly impact the overall quality of trauma care. With the deepening understanding of the aging process, the assessment of frailty becomes more and more sophisticated. It is worth noting that the computational intelligent prediction tools developed from secondary analyses of large number of geriatric trauma patients’ data, are also available for clinical diagnosis and treatment. Further study is needed to determine the best application of these data in clinical practice.

Nevertheless, there are some limitations to this research. This is a single center retrospective research and may not be generalizable. It is prone to lack or miss of data and selection bias. Data on comorbidity, cognitive and motor status/disability, or pre-existing medications are scarce and may change the outcomes. Our study also failed to evaluate the long-term prognosis of the injured patients. Besides, part of the reason for the low mortality rate of injured patients may be that patients die before they arrived at the hospital. And there is no formal regional trauma registration system to ensure the collection of pre-hospital deaths data. The levels of evidence in this field is low, indicating that it is still in the early stages of development. Observational studies are dominant rather than well-constructed randomized controlled intervention trials. More research is needed before further conclusions can be drawn. In spite of these limitations, our research still has important strengths.

## Conclusion

On the whole, healthcare providers and patients may profit from exploring how computational intelligence might improve healthcare. The results of this study provide a snapshot of the trauma burden in a proportion of the urban geriatric patients in Eastern China. The geriatric patients are unlike their younger counterparts, and their unique features should be considered in the future development of computational intelligence, particularly in the case of localized TSFI design. The information gathered using these techniques could also help healthcare providers learn more about their patients' preferences and needs.

## Supplementary Information

Below is the link to the electronic supplementary material.

Supplementary file1 (DOCX 16 KB)

## Data Availability

Data were collected and available at the Department of Emergency Surgery, Wuxi No.9 People’s Hospital affiliated to Soochow University, Wuxi, PR China.

## Abbreviations

AIS: Abbreviated Injury Scale
COVID-19: Coronavirus Disease 2019
ICU: Intensive care unit
ISS: Injury severity score
IQR: Interquartile range
LMICs: Low- and middle-income countries
TSFI: Trauma-Specific Frailty Index
