# Supplementary material for: Overview of Geriatric Trauma in an Urban Trauma Center in Eastern China: Implications from Computational Intelligence for Localized Trauma-Specific Frailty Index System Design
Source: Int J Comput Intell Syst. 2023 Apr 18;16(1):57. doi: 10.1007/s44196-023-00247-0 (PMC10112314; doi:10.1007/s44196-023-00247-0)
Supplement: Supplementary file 1 — Supplementary file1 (DOCX 16 KB) [file 44196_2023_247_MOESM1_ESM.docx]

Supplementary Table 1. Demographic and injury event details of the pre-COVID-19 and COVID-19 cohorts

| Characteristics | Pre-COVID-19 (n=1240) | COVID-19 (n=1354) | *P-*value |
| --- | --- | --- | --- |
| ***Demographic Data*** |  |  |  |
| **Gender** |  |  | 0.619 |
| Female | 418(33.7%) | 469(34.6%) |  |
| Male | 822(66.3%) | 885(65.4%) |  |
| **Age, years** |  |  |  |
| Mean ± SD | 49.7 ±16.0 | 51.4 ±16.3 | **0.007** |
| ***Injury-related Data*** |  |  |  |
| **Trauma type，n（%）** |  |  | 0.096 |
| Blunt | 976 (78.8%) | 1028 (76.0%) |  |
| Sharp | 263 (21.2%) | 324(24.0%) |  |
| **Injuries Sustained，n（%）** |  |  | 0.734 |
| Isolated injury | 853 (69.0%) | 924 (68.4%) |  |
| Multiple body regions | 383(31.0%) | 427 (31.6%) |  |
| **Mechanism of injury, n (%)** |  |  | 0.337 |
| Traffic accident | 464 (37.4%) | 503(37.1%) |  |
| Industrial injury | 448 (36.1%) | 507 (37.4%) |  |
| Assault | 63(5.1%) | 48 (3.5%) |  |
| High fall（≥2m） | 95 (7.7%) | 108 (8.0%) |  |
| Low fall（<2m） | 139 (11.2%) | 159 (11.7%) |  |
| Self-mutilation | 11 (0.9%) | 16(1.2%) |  |
| Other* | 20 (1.6%) | 13(1.0%) |  |
| **Injury Severity Score** |  |  |  |
| ISS, (median, IQR) | 8 (4-9) | 8 (4-9) | 0.055 |
| Major trauma (ISS >15) , n（%） | 140(11.3%) | 139 (10.3%) | 0.406 |
| **Admission to ICU, n（%）** | 56(4.5%) | 40 (3.0%) | **0.035** |
| **Died in 24h, n（%）** | 19 (1.5%) | 20 (1.5%) | 0.908 |

*Includes electrical, chemical, recreational, iatrogenic injuries and so on.
